# The CD147 Protein Complex Is Involved in Entry of Chikungunya Virus and Related Alphaviruses in Human Cells

**DOI:** 10.3389/fmicb.2021.615165

**Published:** 2021-02-25

**Authors:** Lien De Caluwé, Sandra Coppens, Katleen Vereecken, Simon Daled, Maarten Dhaenens, Xaveer Van Ostade, Dieter Deforce, Kevin K. Ariën, Koen Bartholomeeusen

**Affiliations:** ^1^Virology Unit, Biomedical Sciences, Institute of Tropical Medicine Antwerp, Antwerp, Belgium; ^2^Laboratory for Pharmaceutical Biotechnology, University of Ghent, Ghent, Belgium; ^3^ProGenTomics, Ghent, Belgium; ^4^Laboratory of Proteinscience, Proteomics and Epigenetic Signaling, Faculty of Pharmaceutical, Biomedical and Veterinary Sciences, University of Antwerp, Antwerp, Belgium; ^5^Department of Biomedical Sciences, University of Antwerp, Antwerp, Belgium

**Keywords:** alphavirus, chikungunya virus, viral entry, CD147, basigin, envelope protein, affinity purification

## Abstract

Chikungunya virus (CHIKV) is an arbovirus with a global spread and significant public health impact. It is a positive stranded RNA alphavirus belonging to the *Togaviridae* family. However, many questions about the replication cycle of CHIKV remain unanswered. The entry process of CHIKV is not completely understood nor are the associated virus-receptor interactions fully identified. Here, we designed an affinity purification mass spectrometry coupled approach that allowed the identification of factors that facilitate entry of CHIKV in human cells. The identified entry factors were further validated using CRISPR/Cas9. In HEK293T cells we identified the CD147 protein complex as an entry factor for CHIKV. We further showed the involvement of the CD147 protein complex in the replication cycle of related alphaviruses. Interestingly, CD147 contains similar protein domains as the previously identified alphavirus entry factor MXRA8.

## Introduction

Chikungunya virus (CHIKV), an alphavirus from the *Togaviridae* family, is mainly transmitted by the bite of infected female *Aedes* mosquitos, primarily *Aedes aegypti* and *Aedes albopictus* (Mascarenhas et al., 2018). It is a spherical, enveloped, positive stranded RNA virus and is about 60–70 nm in diameter (Solignat et al., 2009). Upon infection, about 85% of the individuals develop chikungunya fever (Burt et al., 2017). After a 3–5 day incubation period, CHIKV infection is characterized by high fever, myalgia, joint pain, headache, rashes and high viremia lasting for several days. Approximately 30–40% of the patients develop chronic chikungunya disease, causing a significant economic burden on society (van Aalst et al., 2017; Paixão et al., 2018). Four different strains exist, West African (WA), Asian, East/Central/South African (ECSA) and Indian Ocean lineage (IOL). The latter emerged from the ECSA lineage in 2005 (Weaver and Forrester, 2015). Currently, neither specific antiviral treatment nor a licensed vaccine is available and treatment of CHIKV infections relies on symptomatic relief (Mascarenhas et al., 2018). Large CHIKV outbreaks in immune-naïve populations are, therefore, associated with a considerable economic burden and the accumulation of medical costs (Mascarenhas et al., 2018).

CHIKV, first described in 1952 in Tanzania (Lumsden, 1955), originally caused only small outbreaks in Africa and Asia (Weaver and Forrester, 2015). This changed drastically in 2004 with the emergence of the novel epidemic IOL CHIKV strain and in the following decades CHIKV was the cause of major outbreaks in sub-Saharan Africa and Asia but also spread to new regions, including Europe and the Americas (Enserink, 2007; Rezza et al., 2007; Grandadam et al., 2011; Kendrick et al., 2014; Delisle et al., 2015; Weaver and Forrester, 2015; Venturi et al., 2017). To date, CHIKV is present in large parts of Africa, Asia, and the tropical regions of the Americas.

The alphavirus genome contains two open reading frames which encode the non-structural and structural proteins, respectively. The genome resembles eukaryotic mRNAs since it possesses a 5′ cap structure and a 3′ poly(A) tail. A subgenomic positive-strand mRNA, referred to as 26S RNA, serves as the mRNA for the synthesis of capsid and the envelope proteins whereas non-structural proteins are directly generated from the genomic RNA (Solignat et al., 2009). Envelope proteins are derived from a structural polyprotein precursor with separate units organized N- to C-terminal as C-E3-E2-6K-E1. E2 and E1 form heterodimers through extensive associations (Voss et al., 2010). The viral envelope complexes are presented on the cell surface as trimers of E2-E1 heterodimers (Solignat et al., 2009; Voss et al., 2010). Each viral particle contains 240 copies of the E2–E1 heterodimer glycoproteins, organized in 80 trimeric spikes arranged in a *T* = 4 icosahedral lattice on the virus surface. Alphavirus attachment and entry into host cells is mediated by these glycoproteins present on the envelope. The E1 and E2 glycoproteins mediate membrane fusion and receptor binding, respectively (Leung et al., 2011).

Generally, enveloped viruses utilize membrane-bound receptors for entry into specific target cells. Previous findings indicate that alphavirus entry is mainly mediated by clathrin-mediated endocytosis (CME) with the requirement of a low endosomal pH and the integrity of the early endosome compartment to productively infect human cells (Sourisseau et al., 2007; Solignat et al., 2009; Bernard et al., 2010; van Duijl-Richter et al., 2015). Binding and infectivity of CHIKV is limited to some cell lines and cellular subpopulations, suggesting that CHIKV is only capable of infecting cells expressing certain surface factors. CHIKV is capable of productively infecting fibroblasts, keratinocytes, melanocytes, epithelial, and endothelial cells and to a lesser extent macrophages (Sourisseau et al., 2007; Matusali et al., 2019). Different experimental approaches have been applied previously to identify entry factors like MXRA8, PHB and ATP5B (Wintachai et al., 2012; Fongsaran et al., 2014; Zhang et al., 2018), responsible for CHIKV binding to and entry in human and mosquito cells. So far only for MXRA8 a direct interaction with the CHIKV envelope proteins was shown, so MXRA8 can be considered as the first bonafide CHIKV receptor (Zhang et al., 2018, 2019; Basore et al., 2019; Song et al., 2019; Kim et al., 2020). However because the previously identified entry factors and receptor (e.g., MXRA8) are not expressed on all susceptible and permissive cell types, the process of CHIKV entry and the receptor complexes engaged are still incompletely understood.

In order to identify unknown host proteins used for CHIKV virus binding and entry, we used an affinity purification coupled to mass spectrometry (AP-MS) approach. We used complete, functional, Strep-tagged envelope complexes (E3-E2-6k-E1) to pull down interactors expressed by target cells.

Here, we identify a new host cell membrane protein complex that is involved in CHIKV entry in human cells. The CD147 protein complex contains a multifunctional transmembrane glycoprotein (CD147, basigin or EMMPRIN), CD98, SLC1A5, ATP1A1, and ATP1B3 (Xu and Hemler, 2005). We show that CD147 has high structural homology with the previously identified CHIKV receptor MXRA8 and demonstrate that the CD147 protein complex is implicated in the entry process of multiple alphaviruses.

## Materials and Methods

### Cells and Viruses

HEK293T cells were cultured at 37°C in Dulbecco’s Modified Eagle medium (DMEM) supplemented with 10% fetal bovine serum (FBS) and 1% L-glutamine. Viral infections were conducted in DMEM medium with 2% FBS and 1% L-glutamine. Following viruses were used: reporter CHIKV with a nanoluciferase gene inserted in nsp3 (Nanoluc CHIKV; ICRES1-P3Nanoluc) (kindely provided by A. Merits), CHIKV (strains: IOL (pBR332-ChikFlic provided by M. Vignuzzi), WA (37997) and Asian (clinical isolate from Aruba-15801654), MAYV (TRVL4675), ONNV (Ahero), SINV (EGAR 339), RRV (National Collection of Pathogenic Viruses catalog number 0005281v), VEEV (TrD), EEEV (H178/99) and WEEV (H160/99). All viruses were propagated on VERO cells except for the reporter CHIKV. Reporter CHIKV stock was created by transfection of viral genomic RNA in HEK293T cells by electroporation. Supernatant was harvested 48 h after transfection. Virus stock was stored at −80°C. Viral genomic RNA was prepared using the SP6 mMessage mMachine transcription kit (Invitrogen) following the manufacturer’s instructions. Plasmid DNA coding for the reporter CHIKV virus carrying a nanoluciferase gene was linearized using *Not*I (Roche), purified by ethanol precipitation and used as template in the transcription reaction. After the reaction was stopped, the transcribed RNA was purified by Lithium Chloride precipitation.

### Affinity Purification and Mass Spectrometry

**FIGURE 1:**
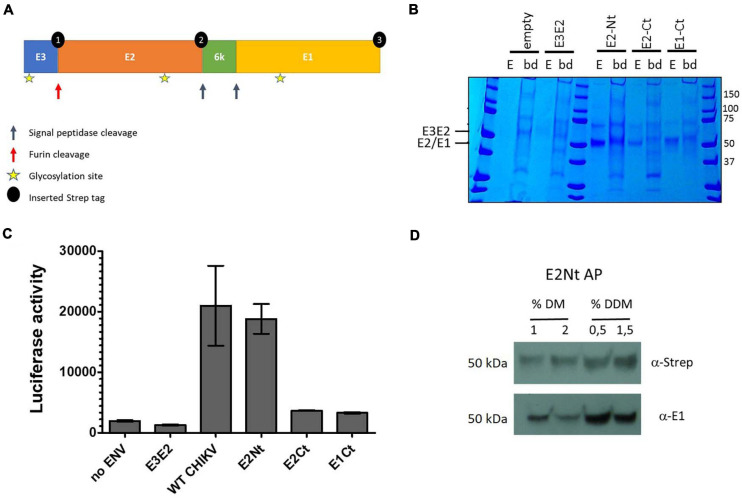
Tagging strategy and affinity purification of internally Strep-tagged CHIKV envelope subunits. **(A)** CHIKV envelope glycoproteins and tagging strategy. Internal 2x Strep-tags were inserted into the full-length envelope gene at three sites (black dots) in separate constructs (E2-Nt = 1, E2-Ct = 2, E1-Ct = 3). **(B)** Internally tagged CHIKV envelope constructs were expressed in HEK293T cells and affinity purified from lysate using Strep-Tactin (position of the tags is according to **(A)**: E2-Nt = 1, E2-Ct = 2, E1-Ct = 3). Cells expressing Strep-tagged E3-E2 (E3-E2) and untransfected (empty) cells were included as negative controls. The eluates (E) of the affinity resin were separated by SDS-PAGE and analyzed by coomassie staining alongside the background protein binding to the beads after elution (bd). **(C)** Infectivity of luciferase reporter pseudovirus expressing WT CHIKV envelope proteins or the three differentially internally tagged constructs. The HEK293T cells were infected for 24 h and luciferase activity measured. Mean ± SD is shown (*n* = 3; 1 independent experiment with three technical replicates). **(D)** Affinity purification of N-terminal Strep-tagged E2 in the presence of 1 or 2% n-decyl-β-D-maltopyranoside (DM) and 0.5 or 1.5% n-dodecyl-β-D-maltopyranoside (DDM). Recuperation of E2 (α-Strep) and E1 (α-E1) is shown.

The full-length CHIKV envelope (E3-E2-6k-E1) gene or capsid was cloned in the mammalian expression vector pcDNA4TO and internal Strep-tags were introduced as indicated in Figure 1A (constructs: pcDNA4/TO-Env-E2-Nt-Strep, pcDNA4/TO-Env-E2-Ct-Strep, pcDNA4/TO-Env-E1-Ct-Strep, pcDNA4/TO-CAP-Nt-Strep). 12 × 10^6^ HEK293T cells were plated and the following day transfected with 20 μg plasmid DNA. Forty eight hours after transfection cells were harvested manually and washed two times with phosphate-buffered saline (PBS). Cells were lysed in 0.5% DDM (Anatrace) in PBS complemented with protease inhibitors (Roche) after which they were sonicated. Cell lysate was centrifuged at 4°C 2,465 g for 5 min. Hereafter supernatant was incubated for 1–2 h on Streptavidin Sepharose High Performance affinity beads (GE Healtcare) on an overhead rotor. Beads were washed three times with 0.5% DDM in PBS. Additional cell lysate of HEK293T cells was prepared in 0.5% DDM in PBS. Beads were incubated with additional cell lysate from HEK293T cells at 28°C for 30 min on an overhead rotor. Beads were washed two times with 0.5% DDM in PBS and one time with trypsin buffer (20 mM Tris.HCl pH 8.0, 2 mM CaCl_2_). The beads were resuspended in 50 μl trypsine buffer and stored at −20°C until further processing. The samples were digested by adding 0.5 μg of trypsin/LysC (Promega) followed by incubation at 37°C overnight. The day after, the samples were centrifuged at 16.000 g and the supernatant was vacuum dried to be resuspended in a 0.1% FA solution prior to LC-MS/MS analysis on a Synapt G2-Si mass spectrometer (Waters) coupled to a Nano-Acquity UPLC liquid chromatography system (Waters). To separate the proteome, samples were first loaded onto a pre-column (180 μm × 20 mm nanoACQUITY UPLC 2G-V/MTrap 5 μm Symmetry C18, Waters) for 5 min at a flowrate of 8 μL/min, 99.5% Solvent A, followed by a 60 min linear gradient elution at a flowrate of 300 nl/min on the analytical column (75 μm × 250 mm ACQUITY UPLC 1.8 μm HSS T3, Waters) from 1 to 40% Solvent B. Solvent A and B consisted of 3% DMSO, 0.1% Formic Acid in UPLC water and 0.1% Formic Acid in acetonitrile, respectively. MS and MS/MS spectra were acquired from 50 to 5,000 m/z in positive mode using High-Definition Data dependent acquisition. Accumulation time was set to 200 ms for MS survey scans, followed by CID fragmentation of up to 12 precursor ions with charge 2, 3 and 4 +. The intensity threshold was set to 3,000 and the collision energy was ramped from 6/9 V (50 m/z, start/end) up to 147/183 V (5,000 m/z, start/end). Tandem mass spectra were accumulated for 100 ms or 100,000 cps with a maximum accumulation time of 250 ms. Dynamic exclusion of fragmented precursor ions was set to 10 s. During ion mobility separation, wave velocity was ramped from 2,500 to 400 m/s, which enabled wideband enhancement, increasing sensitivity for singly charged fragment ions. In parallel, lockspray of glufibrinopeptide-B (m/z 785.8427) was acquired for 200 ms at a scan frequency of 60 s for mass calibration purposes (Helm et al., 2014). Data was analyzed using the MiST algorithm (Verschueren et al., 2015).

### Pseudovirus

Pseudoviral particles were produced in HEK293T cells. The cells were seeded 24 h before transfection and co-transfected with the plasmids coding a lentiviral reporter pNL4-3-Luc R-E- and pcDNA4/TO-Env, pcDNA4/TO-Env-E2-Nt-Strep, pcDNA4/TO-Env-E2-Ct-Strep, pcDNA4/TO-Env-E1-Ct-Strep or pcDNA4/TO-Cter2xStrep-E3E2 using Fugene 6 (Promega). Forty eight hours post transfection pseudoviral particles were harvested and filtered (0.45 μm).

To check the functionality of the different constructs, pseudoviral particles were allowed to infect HEK293T cells for 48 h were after luciferase activity was measured on TriStar LB 941 Multimode Microplate Reader (Berthold Technologies).

### gRNA Cloning

gRNA’s were designed using CHOPCHOP^1^ and cloned in pSpCas9(BB)-2A-Puro (PX459) V2.0 (a gift from Feng Zhang; Addgene plasmid # 62988; Ran et al., 2013) carrying Cas9, a gRNA scaffold and an ampicillin and puromycin resistance gene. pSpCas9(BB)-2A-Puro (PX459) V2.0 was linearized using *Bbs*I (New England Biolabs). The linearized plasmid was purified in gel using Wizard^®^ SV Gel and PCR Clean-Up (Promega) according to the manufacturer’s protocol. Top and bottom strands of the gRNA’s oligo’s were annealed on a thermocycler using following parameters: 95°C for 1 min; ramp down to 20°C at 5°C/30 sec. The annealed gRNA’s were ligated in the linearized pSpCas9(BB)-2A-Puro (PX459) V2.0 using T4 ligase (Roche) at room temperature for 30 min. The ligated plasmids were transformed in Stbl3 cells (Invitrogen) using a heat shock at 42°C. The right gRNA insertion in the plasmids was checked by Sanger sequencing.

A second gRNA [CD147(b) or CD98(b)] was inserted in pSpCas9-SLC1A5(2)(b)-2A-Puro so that the plasmid carried two gRNA’s. This to allow simultaneous KO of two genes. The gRNA’s and their scaffold were amplified from pSpCas9-CD147(b)-2A-Puro and pSpCas9-CD98(b)-2A-Puro. Following primers carrying *Xba*I restriction sites gRNA_f ATCTAGAGAGGGCCTATTTCCCATGATTCC and gRNA_R ATCTAGACGCGCTAAAAACGGACTAGC were used. The PCR products were purified in gel were after the PCR products and plasmid were digested with *Xba*I (Roche). To prevent religation the digested plasmid was dephosphorylated using Shrimp alkaline phosphatase (Roche). The PCR products were ligated in the linearized, dephosphorylated plasmid using T4 ligase at room temperature for 6 h. The plasmids were transformed in Stbl3 cells using a heat shock at 42°C. Right insertion of the second gRNA’s was controlled with Sanger sequencing.

### CRISPR/Cas9 Knockout Screen

A total of 600,000 HEK293T cells were plated in a 6 well plate. Sixteen and twenty four hours after plating, the cells were transfected with 1 μg of the gRNA containing constructs using fugene 6 (Promega). Eighteen and twenty four hours after transfection cells were harvested and split in three wells of a 6 well plate. Sixteen and twenty four hours after splitting, selection with 1.3 μg/ml puromycin (Sigma) was started for at least 72 h or until untransfected control cells were completely killed by the puromycin. Selected cells were harvested manually and replated in a 24 well plate in the presence of 0.5 μg/ml puromycin. The following day the transient knockout cells were infected with reporter CHIKV harboring a nanoluciferase gene. A 1 × 10^–3^ dilution of the reporter virus stock was used. This dilution allows detection in the linear phase of the luciferase signal (Supplementary Figure 1). Lower dilutions could not be used since this leads to an strong increase of variability between the technical replicates. Twenty four hours after infection nanoluciferase activity was measured using Nano-Glo^®^ Luciferase Assay System (Promega) on the TriStar LB 941 Multimode Microplate Reader. To bypass the entry pathway, the transient KO cells were transfected with 0.5 μg RNA/24 well of the reporter CHIKV using TransIT-mRNA (Mirus). Six hours after RNA transfection luciferase activity was measured.

### Western Blot

Cell lysates were prepared in 4x loading buffer 200 mM Tris, pH 6.8, 8% SDS, 40% glycerol and 0.02% bromophenol blue under reducing conditions (+ dithiothreitol) and heated to 80°C for 10 min. Samples were separated by SDS-PAGE and transferred to a nitrocellulose membrane using the Trans-Blot Turbo Transfer System (Bio-Rad). Membranes were blocked in 5% powdered milk in PBS-T (0.05% Tween-20 in PBS) for 1 h at room temperature with constant agitation. Membranes were washed with PBS-T and incubated with primary antibody anti-CD147 (ab232967, Abcam), anti-ASCT2 (SLC1A5) (V501, Cell Signaling), anti-CD98 (12206-T62, sino biologicals), anti-E1, anti-Strep (ab184224 or ab180957, Abcam) and anti-E2 (NR44002, Bei Resources) overnight at 4°C. After washing with PBS-T, the membranes were incubated the secondary antibody anti-mouse (ab6728, Abcam) or anti-rabbit (ab6721, Abcam) for 1 h at room temperature. Detection was done with the Amersham ECL Western Blotting Detection Reagent (GE healthcare).

### Stable KO Cell Lines

KO cells were transfected as described as above. After selection with 1.3 μg/ml puromycin, cells were harvested and diluted to a concentration of 0.5 cells per 100 μl. The cell suspension was plated in a 96 well plate (100 μl/well). Cells were expanded for 2–3 weeks and transferred to a 24 well when they were 50% confluent. KO efficiency was checked using Western blot.

For infectivity assays, stable KO cell lines were infected with the same reporter CHIKV dilution as in the CRISPR/Cas9 screen (Supplementary Figure 1), for 24 h were after luciferase activity was measured.

### CD147 Membrane Staining

Wild-type (WT) and KO cells were harvested manually and washed two times with PBS/BSA. The cells were incubated with the primary anti-CD147 antibody (ab232967, Abcam) (1 μg/ml) for 30 min at room temperature. Subsequently they were washed three times with PBS/BSA and fixed with 1% paraformaldehyde in PBS for 15 min at room temperature. Cells were washed again two times with PBS/BSA and incubated with the secondary antibody (A21070, Invitrogen) (0.5 μg/ml) for 30 min at room temperature. Lastly the cells were washed three times with PBS/BSA and analyzed on FACSVerse (BD).

### Reintroduction of CD147 and SLC1A5

Expression constructs for CD147 and SLC1A5, pLX304-BSG and pLX304-SLC1A5, were provided through DNASU (DNASU # HsCD00442351 and # HsCD00436374) [50]. The construct were mutated at the gRNA target site to prevent cutting by Cas9 using the QuikChange Lightning Site-Directed Mutagenesis Kit (Agilent Technologies) according to the manufacturer’s protocol. Following primers CD147mut_f GAGTGAAGGCTGTGAAGTC AAGCGAACACATCAACGAGGGGG, CD147mut_r CCCCCT CGTTGATGTGTTCGCTTGACTTCACAGCCTTCACTC, SLC 1A5mut_f GCAAAAACCCCTACCGCTTTTTATGGGGCATC GTGACGCCGC and SLC1A5_r GCGGCGTCACGATGCCCC ATAAAAAGCGGTAGGGGTTTTTGC were used.

The day before transfection, 35,000 cells of the different KO cell lines were plated in 96 well plate. One hundred nanogram of pLX304-BSGmut or pLX304-SLC1A5mut was transfected in the different KO cell lines using fugene 6. Forty eight hours after transfection, cells were infected with reporter CHIKV, using the same dilution as in the CRISPR/Cas9 screen (Supplementary Figure 1), and luciferase activity was measured as described above.

### Envelope Protein Staining

A total of 35,000 stable KO cells were plated in a 96 well plate the day before infection. Stable KO cells were infected with CHIKV [Nanoluc (28 h), WA (28 h), Asian (52 h) and IOL (48 h), RRV (48 h), ONNV (48 h), MAYV (48 h), SINV (24 h), EEEV (28 h), VEEV (28 h) and WEEV (24 h). All virus stocks were titrated on wild-type cells to determine a MOI of 1. Assays were performed using 10-fold dilutions of this MOI.

Infected cells were harvested manually and washed two times with PBS. Cells were fixed with 1% paraformaldehyde in PBS for 15 min at room temperature. Subsequently they were washed two times with PBS + 1% BSA + 0.05% sodium azide (PBS/BSA). Cells were permeabilized in 0.5% Tween-20 in PBS for 15 min at room temperature. Next, the cells were washed one time in 0.1% Tween-20 in PBS/BSA (PBS/BSAT). Incubation with the primary antibody (NR44002, Bei Resources or CK119; Tuekprakhon et al., 2018) (1 μg/ml) diluted in PBS/BSAT at 4°C was done overnight. Cells were washed three times with PBS/BSAT and incubated with the secondary antibody (A11001, Invitrogen) (0.5 μg/ml) for 1 h at 4°C. Finally the cells were washed again three times with PBS/BSAT and resuspended in PBS/BSA. Cells were analyzed on FACSVerse (BD).

### Binding Assay

A total of 800,000 HEK293T or HEK293T ΔCD147 cl1 were plated in a 6 well plate. The next day cells were incubated with Nanoluc CHIKV (MOI of 2) for 45 min at 37°C. Cells were washed four times in DMEM medium with 2% FBS and 1% L-glutamine. After washing, the remaining cells were counted. Cells were pelleted and resuspended in 1 ml TRIzol and RNA was purified. Viral genomes were quantified using RT-qPCR. Ct–values were transformed to linear scale expression quantities and were normalized for number of cells.

### Statistical Analysis

GraphPad Prism or SPSS software was used to conduct statistical analysis and graphing. A one-way analysis of variance (ANOVA) with Bonferroni *post hoc* testing was used for comparing the luciferase activity in the different KO cell lines after infection with CHIKV Nanoluc. For the reintroduction assay and the flow cytometric assay analyzing the different alphaviruses a two-way ANOVA with Bonferroni *post hoc* testing was used. The CRISPR/Cas9 screen groups were evaluated using Dunnett’s test for multiple comparison and by comparing to the standard deviation of the control group. A *t*-test was used to determine significance in the binding assay.

## Results

### Creation of a Functional Strep-Tagged CHIKV Envelope Gene

The CHIKV envelope is produced as a polyprotein precursor containing E3-E2-6K-E1. Targeting of the transmembrane envelope complex to the endoplasmic reticulum and Golgi complex is required for correct post-translational modification by proteolytic processing and glycosylation. Oligomerization of the E2-E1 subunits is further required for correct folding and stabilization of these subunits and thus necessary for optimal binding of the envelope to its cellular receptors. To allow transient expression of the native envelope complex, we introduced internal Strep-tags in the full-length envelope gene (E3-E2-6k-E1), derived from a IOL strain carrying the E1 A226V mutation (Figure 1A). The internal Strep-tags were introduced taking care to retain furin and signalpeptidase recognition sites for proper subunit proteolytic processing, resulting in three separate tagged constructs [E2 N-terminal tagged (E2-Nt), E2 C-terminal tagged (E2-Ct) and E1 C-terminal tagged (E1-Ct)] (Figure 1A). Affinity purification allowed efficient recuperation of all three tagged envelope proteins (E2-Nt, E2-Ct, and E1-Ct) (Figure 1B). Correct proteolytic processing of envelope pro-proteins was evidenced by the migration of the separated envelope subunits, E1 and E2, at their expected molecular weights (E2 and E1 expected MW is 47 kDa) (Figure 1B). Since the introduction of an internal Strep tag could interfere with protein folding and receptor binding, the functionality of these three constructs was tested using a lentiviral pseudovirus infectivity assay. We found that the E2-Nt tagged construct had an equivalent infectivity compared to the WT CHIKV envelope (Figure 1C), indicating its capacity to fully engage its cellular receptor(s). The fully functional E2-Nt tagged construct E3-ST-E2-6k-E1 was therefore used in all further experiments.

Affinity purification of E3-ST-E2-6k-E1 was optimized using detergents that allow extraction of intact and functional membrane associated complexes (Laganowsky et al., 2013). Several concentrations of n-Dodecyl β-D-maltoside (DDM) or n-decyl-β-D-maltopyranoside (DM) were compared for allowing the recuperation of intact E2-E1 oligomers. Using a lysis buffer containing 0.5% DDM, the E2-E1 oligomer formation of the envelope proteins is best maintained during purification, evidenced by de recuperation of E1 after affinity purification of the Strep-tagged E2 (Figure 1D). Therefore in all further experiments a lysis buffer containing 0.5% DDM was used.

Importantly, the primary IOL strain derived envelope gene used here does not contain mutations associated with increased glycosaminoglycan (GAG) dependency e.g., E79K, G82R, and E166K (Henrik Gad et al., 2012; Ashbrook et al., 2014; Silva et al., 2014; Weber et al., 2017). This presumably precludes enhancement on GAG mediated cell attachment as a mechanism for infection and increases the likelihood of identifying proteinaceous entry factors.

### Identification of E-Protein Interactors Using Affinity Purification Followed by Mass Spectrometry

**FIGURE 2:**
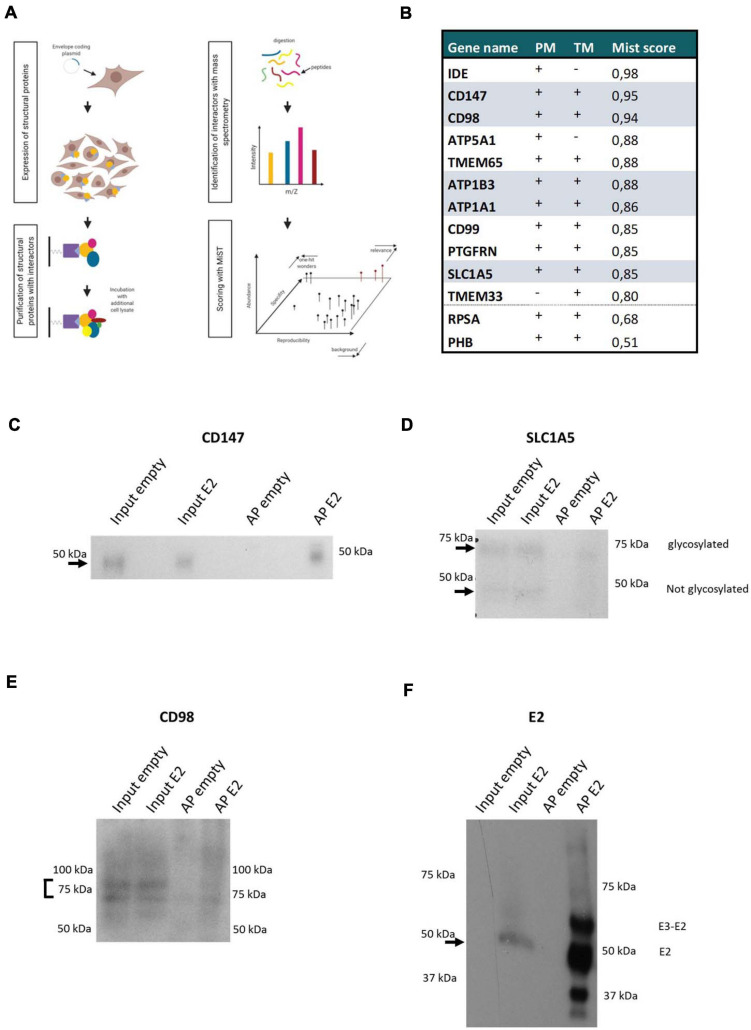
CHIKV envelope association with the CD147 complex **(A)** Scheme of the used AP-MS approach. **(B)** CHIKV envelope Interaction partners of interest are listed. Examination of their plasma membrane presence (PM), presence of a transmembrane domain (TM), and MiST score (MiST) are indicated. Components of the CD147 protein complex retrieved in the CHIKV envelope affinity purification are indicated. **(C–F)** Western Blot of affinity purification of Strep-tagged Envelope protein. Detection of E2 and the interactors CD147, CD98, and SLC1A5. Affinity purification of untransfected cells (empty) were included as controls. On each blot input lysates of untransfected cells (empty) or E2 expressing cells (E2) and affinity purifications (AP) of the empty or E2 cells were loaded. For the CD147, CD98 and SLC1A5 blots 0.4 μl of input lysates and 10 μl of APs was loaded. For the E2 blot 5 μl of input lysates and 10 μl of APs was loaded.

To identify entry factors required for CHIKV infection, affinity purification of the Strep-tagged E3-ST-E2-6k-E1 was performed. Envelope proteins were produced in HEK293T cells, purified using Strep-affinity resin and additionally incubated with lysate of human cells (HEK293T). After washing, on-bead digestion with trypsin was performed to minimize complex disruption and ensure maximum recovery of associated proteins (Turriziani et al., 2014). The purifications were performed in triplicate alongside triplicate control purifications using untreated HEK293T cells and compared with affinity purification of the Strep-tagged capsid protein to eliminate non-specific protein binding. The most specific and abundant interaction partners of the CHIKV envelope proteins were identified using MiST (mass spectrometry interaction statistics) (Figure 2A; Verschueren et al., 2015).

In total, 730 proteins were identified of which 39 proteins had a MiST score higher than 0.8. The candidate proteins were examined for the presence of transmembrane regions and their known expression on the plasma membrane by literature search, database mining (uniprot, COMPARTMENTS and the human protein atlas) and prediction tools (TMpred) (Figure 2B). Of these 39 proteins, 11 proteins were selected for further functional validation based on their subcellular localization at the plasma membrane, relevant interactions or complexes, or their known involvement in the entry process of other (alpha)viruses (Figure 2B). Interestingly, five components (CD98, CD147, SLC1A5, ATP1A1, and ATP1B3) of a previously described protein complex were identified with a MiST score above 0.8 (Xu and Hemler, 2005). The identification of this protein complex after affinity purification was validated on Western Blot (Figures 2C–F). ATP1A1 was previously suggested to play a role in the entry process of CHIKV (Ashbrook et al., 2016). We further found prohibitin (PHB) (MiST score = 0.51) which was identified previously as an entry factor for CHIKV virus in a virus-overlay-assay (Wintachai et al., 2012). ATP5B, ATP synthase subunit b, was previously identified with a virus-overlay-assay in mosquito cells (Fongsaran et al., 2014). Here with our strategy, we identified ATP5A1, another subunit of the ATP synthase complex. In addition we identified RPSA (Mist score = 0.68) which was suggested to facilitate entry of SINV, Venezuelan equine encephalitis virus (VEEV), dengue virus, adeno-associated virus, West Nile virus, tick-borne encephalitis virus and Japanese encephalitis virus (DiGiacomo and Meruelo, 2016). The diversity of viruses for which RPSA has been implicated as a potential entry-related factor, rather suggests a role as attachment factor instead of a specific entry receptor. Because of their previous implications in the virus entry process, both PHB and RPSA were included in subsequent validation experiments despite their lower MiST score. Altogether, this confirms that our approach is capable of identifying valuable protein-protein interactions which potentially contribute to the entry process of CHIKV.

### CRISPR/Cas9 Knockout Screen Identifies Components of the CD147 Complex as Potential CHIKV Entry Factors

**FIGURE 3:**
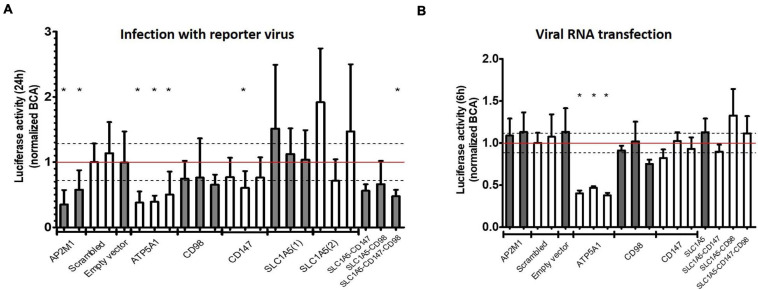
Validation of identified receptor candidates with CRISPR-Cas9 knockout. **(A,B)** Knockout screen of identified human receptor candidates. HEK293T cells were separately transiently transfected with three different gRNA’s per gene and transfected cells selected with puromycin. As a positive control AP2M1 and as a negative control scrambled and Empty vector (Cas9 vector not containing gRNA) were included. Data was normalized for the scrambled control. Dashed lines depict standard deviation for scrambled. **(A)** Infection with reporter virus (Nanoluc CHIKV) containing nanoluciferase for 24 h (entry dependent). **(B)** Transfection with viral genomic RNA for 6 h (entry independent). Mean ± SD is shown (*n* = at least 6). For each construct at least 2 independent experiments with 3 technical replicates were performed. * *p* < 0.05.

To further validate the membrane proteins as possible receptors of CHIKV, we knocked out the selected proteins individually using transient CRISPR/Cas9 transfection. Three different guide RNA’s (gRNA’s) were designed for each gene to ensure effective gene targeting and prevent off-target effects of CRISPR/Cas9 knockout. Two separate sets of three gRNA’s for SLC1A5 were designed to allow knockout of two separate isoforms of SLC1A5 which could not be targeted simultaneously. To allow simultaneous knockout of two genes of the identified CD147 protein complex, a second gRNA (targeting CD147 or CD98) was cloned in the expression construct containing the gRNA for SLC1A5 (yielding SLC1A5-CD147 and SLC1A5-CD98 constructs). Co-transfection of these two latter constructs, targeted three different proteins (SLC1A5-CD147-CD98) of the CD147 protein complex simultaneously. As a control for the role of CME in CHIKV entry and infectivity, the critical CME cofactor AP2M1 (McMahon and Boucrot, 2011) was also depleted by CRISPR/Cas9. As an additional control NRAMP2, a proposed cellular receptor for the related SINV (Rose et al., 2011) was similarly depleted by CRISPR/Cas9. Knockout efficiency of CD147, CD98, and SLC1A5 after transient transfection was visualized with Western Blot (Supplementary Figure 2). HEK293T knockout cells were enriched by puromycin selection after which they were infected with an IOL CHIKV clone containing a nanoluciferase reporter gene (Nanoluc CHIKV) to assess infectivity (Figure 3A and Supplementary Figure 3). Factors related to the CD147 complex and those with an impact on CHIKV entry are depicted in Figure 3A. The entire knockout screen with all identified factors is shown in Supplementary Figure 3. To rule out non-entry related effects of the depletion of the targeted genes, knockout cells were transfected with the viral genomic RNA carrying the nanoluciferase reporter gene (Figure 3B).

Depletion of AP2M1 resulted in decreased viral infectivity after infection but not after transfection with viral RNA which confirms the involvement of CME in the entry process of CHIKV (Figures 3A,B). In contrast, as knockout of ATP5A1 impaired infectivity of CHIKV potently, a similar decrease in viral replication was seen after viral RNA transfection, ruling out a role for ATP5A1 in the viral entry process (Figures 3A,B). Knockout of PHB or RPSA, previously implicated in entry processes of CHIKV or related viruses, presented an inconsistent and limited effect on viral replication, while knockout of NRAMP2, previously shown to support entry of SINV, did not interfere with viral replication of CHIKV (Supplementary Figure 3).

While KO of separate components of the CD147 complex suppressed infectivity of CHIKV only mildly (Figure 3A, CD147 and CD98) or had no effect (Figure 3A, SLC1A5), the combined depletion of two factors of the CD147 complex (SLC1A5 and CD147 or SLC1A5 and CD98) led to a decrease in viral infectivity, similar to depletion of the critical CME component AP2M1 (Figure 3A). Importantly, combined KO of factors of the CD147 complex did not interfere with viral replication after transfection of viral RNA (Figure 3B). To control for different growth rates between the transient KO cells, all samples were normalized for their protein concentration measured by a bicinchoninic acid (BCA) assay.

All together, these data clearly suggest a role for the CD147 protein complex in the replication process of CHIKV in human cells. The CD147 protein complex is involved in a step prior to viral RNA synthesis, evidenced by the viral RNA transfection assay.

### Stable Clonal KO Cell Lines and Reintroduction of CD147 Complex Components Suggest CD147 as Main Determinant in CHIKV Entry

The different factors in the CD147 complex stabilize each other’s plasma membrane presentation and orientation (Xu and Hemler, 2005; Guo et al., 2015), which is probably important in virus entry. Indeed, while simultaneous depletion of multiple components of the CD147 complex allowed us to implicate this complex in viral entry, the incomplete depletion of individual proteins by the polyclonal CRISPR/Cas9 protocol precluded identification of a main determinant for CHIKV entry (Figure 3A and Supplementary Figure 2).

To ensure complete depletion and to assess the role of individual members of the CD147 complex in cellular entry of CHIKV, stable KO HEK293T cell lines harboring knockouts of CD147, SLC1A5, and SLC1A5-CD147 combined were created. Depletion of total cellular protein levels was validated on Western Blot (Supplementary Figure 4) and the membrane expression of CD147 was assessed with flow cytometry (Supplementary Figure 5). For the double KO cell line HEK293T ΔCD147-SLC1A5 cl2 some residual CD147 membrane expression is observed.

**FIGURE 4:**
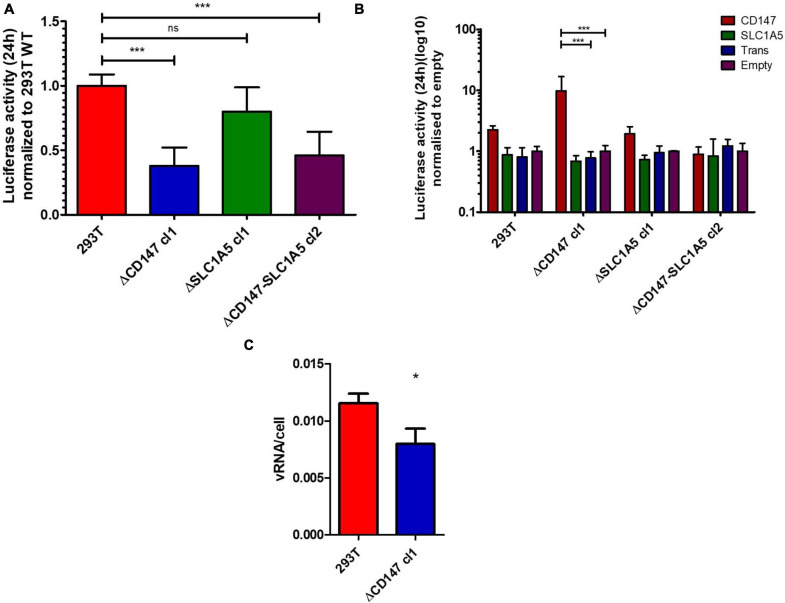
Effect of CD147 expression on CHIKV infection efficiency. **(A)** Stable knockout cell lines of CD147, SLC1A5 and CD147-SLC1A5 combined were infected with a reporter Nanoluc CHIKV for 24 h. Data is normalized to 293T WT. Mean ± SD is shown (*n* = 6; 2 independent experiments with 3 technical replicates). **(B)** Reintroduction of CD147 or SLC1A5 in the stable KO cell lines used in **(A)**. Expression constructs had synonymous mutations at the target gRNA site. Both a transfection control (trans) and a no transfection control (empty) were used. Normalization with the no transfection control (empty) within each KO cell line. Mean ± SD is shown (*n* = 3; 3 independent transfections) **(C)** Binding assay with Nanoluc CHIKV on HEK293T and HEK293T ΔCD147 cl1 cells. Cell associated viral RNA was measured after 45 min virus binding at 37°C using RT-qPCR. Mean ± SD is shown (*n* = 3; 1 experiment with 3 technical replicates) *p < 0.05, ***p < 0.001.

The infection rate of the reporter virus was significantly decreased in KO cell lines devoid of CD147 (Figure 4A, HEK293T ΔCD147 cl1 and HEK293T ΔCD147-SLC1A5 cl2) but relatively unimpaired in cells lacking SLC1A5 (Figure 4A, HEK293T ΔSLC1A5 cl1). To exclude off-target effects in the monoclonal KO cell lines, CD147 and SLC1A5 were reintroduced by transient transfection of CRISPR/Cas9 insensitive expression vectors (Figure 4B).

CD147 is widely expressed in a short isoform 2, while expression of its longer isoform 1 is restricted to the retina (Muramatsu, 2016). CD147 isoform 2 and SLC1A5 were both reintroduced in the KO cell lines (Figure 4B and Supplementary Figure 6). Depending on the glycosylation, CD147 migrates between 31 and 65 kDa on a Western blot (Grass and Toole, 2016). After reintroduction, the majority of overexpressed CD147 is low glycosylated while also a minority of high glycosylated, similar to the endogenous form of CD147 in HEK293T cells, is found. Reintroduction of CD147 isoform 2 stimulated infectivity in HEK293T WT cells and restored infectivity in HEK293T ΔCD147 cells (Figure 4B), while reintroduction of SLC1A5 in WT or HEK293T ΔSLC1A5 cells did not stimulate infectivity (Figure 4B). Interestingly, reintroduction of CD147 in cells that were depleted of SLC1A5 or both CD147 and SLC1A5 also showed limited or no reconstitution of infectivity (Figure 4B).

To assess whether the absence of CD147 impacted the association of viral particles with the cell a binding assay was performed. WT and CD147 KO cells were incubated for 45 min with the same reporter virus at an MOI of 2 and extensively washed. Determination of viral RNA associated with the respective cells indicated that less virus was stably associated with cells lacking CD147 (Figure 4C).

Together these data strongly suggest a role for CD147 in CHIKV entry as the main interaction determinant. They further suggest the need for other components like SLC1A5 in the complex to be present to allow sufficient or correct expression of CD147 on the plasma membrane to allow its functioning in virus entry.

### Role for CD147 Protein Complex in Replication of Related Alphaviruses

Four different lineages of CHIKV have been described, i.e., ECSA, IOL, WA, and Asian. The reporter virus used in the KO screen is derived from an IOL clone. To further generalize our observation, we investigated the role of the CD147 protein complex in the entry of Asian, WA and IOL CHIKV as well as in the entry of the related alphaviruses o’nyong’nyong virus (ONNV), Mayaro virus (MAYV), Ross River virus (RRV), Sindbis virus (SINV), Eastern equine encephalitis virus (EEEV), Venezuelan equine encephalitis virus (VEEV), and Western equine encephalitis virus (WEEV). The CHIKV strains and alphaviruses were analyzed on stable KO cell lines harboring knockouts of CD147, SLC1A5, and SLC1A5-CD147 combined. After infection of the knockout cells with Asian CHIKV (52 h), ONNV (48 h), MAYV (48 h), CHIKV Nanoluc (28 h), WA CHIKV (28 h), IOL CHIKV (48 h), RRV (48 h), SINV (24 h), EEEV (28 h), VEEV (28 h), and WEEV (24 h), cells were stained for envelope (E) proteins and analyzed with flow cytometry. All virus stocks were titrated on wild-type cells to determine a MOI of 1. Assays were performed using 10-fold dilutions of this MOI.

**FIGURE 5:**
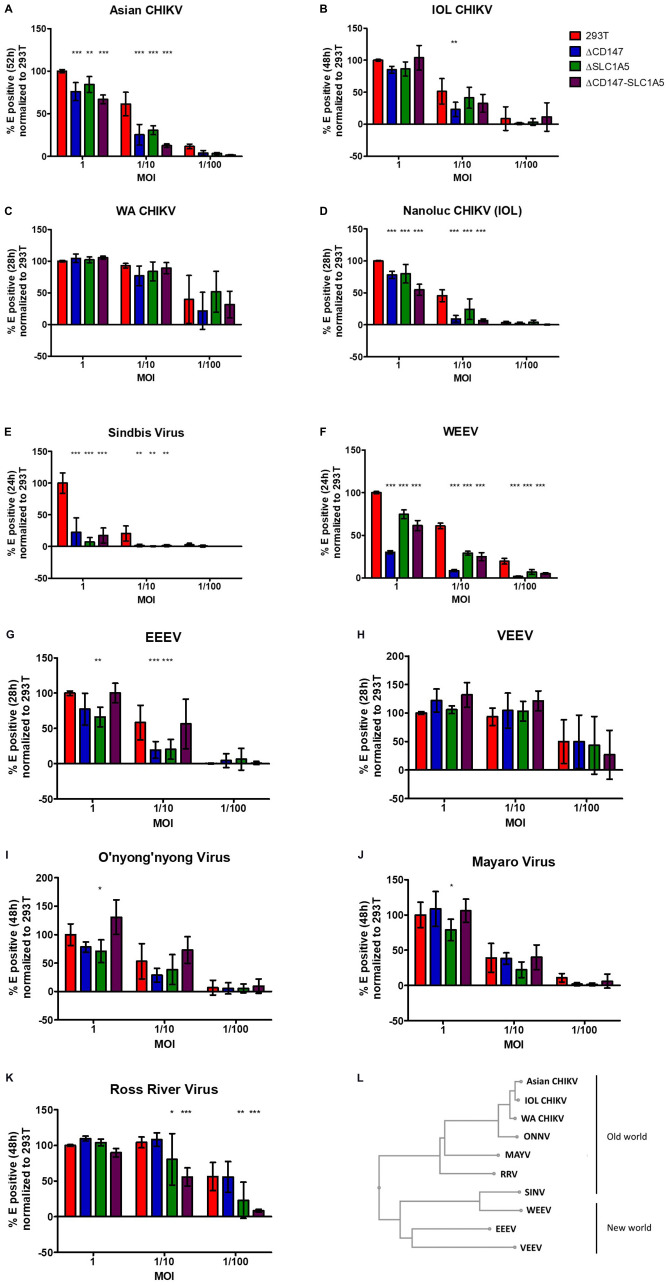
The role of the CD147 protein complex in different CHIKV strains and related alphaviruses. **(A–K)** stable KO cell lines defective of CD147 or SLC1A5 or both CD147 and SLC1A5 were used **(A)** infection with Asian strain CHIKV for 52 h **(B)** infection with IOL for 48 h **(C)** infection with WA CHIKV for 28 h. **(D)** infection with CHIKV carrying a reporter nanoluciferase gene for 28 h **(E)** infection with SINV for 24 h **(F)** infection with WEEV for 24 h **(G)** infection with EEEV for 28 h **(H)** infection with VEEV for 28 h **(I)** infection with ONNV for 48 h **(J)** infection with MAYV for 48 h **(K)** infection with RRV for 48 h. Detection of infected KO cells with E staining combined with flow cytometry. All graphs depict percentage E positive cells. **(A–K)** All graphs were normalized to the percentage E-positive WT HEK293T cells in highest virus concentration. Mean ± SD is shown (*n* = 6; 2 independent experiments with 3 technical replicates); *p < 0.05, **p < 0.01, ***p < 0.001 compared to WT HEK293T cells **(L)** Phylogenetic tree of Old and New world alphaviruses based on E2.

The different alphaviruses were tested on stable KO cell lines HEK293T ΔCD147 cl1, HEK293T ΔSLC1A5 cl1 and HEK293T ΔCD147-SLC1A5 cl2 (Figures 5A–K). Too high virus numbers will lead to a generalized infection whereas too low viral numbers may increase the variability of the assay. When the reporter CHIKV (Nanoluc CHIKV) was used to infect the stable KO cell lines, a similar effect of CD147 and CD147-SLC1A5 knockout (Figure 5D) was seen evidencing equivalence of both the reporter virus assay (Figure 4A) and the flow cytometry based assay (Figure 5D) to detect a role for the CD147 protein complex in the CHIKV replication cycle. The effect of the CD147 protein complex was further analyzed for Asian CHIKV, WA CHIKV, and IOL CHIKV (Figures 5A–C). For Asian CHIKV a significant decrease in viral replication was observed when high or intermediate viral concentrations were used on all three KO cell lines (Figure 5A). A clear negative effect on viral replication of IOL CHIKV was seen with intermediate amounts of virus in HEK293T ΔCD147. A trend on HEK293T ΔCD147 cells was visible when low amounts of virus were used (Figure 5B). With WA CHIKV only a trend is noticeable with the lower amount of virus when CD147 or CD147-SLC1A5 are simultaneously knocked out and this due to a higher observed variability in the assay for this virus.

Almost no viral replication was detected in the different KO cell lines when they were infected with SINV compared to wild-type HEK293T cells (Figure 5E). For the New World alphavirus WEEV, which derived its structural proteins from a SINV ancestor, also a significant effect on virus replication was seen in the three different KO cell lines and this with all the tested virus dilutions (Figure 5F). For the New World alphavirus EEEV an effect on virus replication was observed in HEK293T ΔCD147 and HEK293T ΔSLC1A5 cells but not in the double KO cell line HEK293T ΔCD147-SLC1A5 (Figure 5G). We observed no role of the CD147 protein complex in the replication cycle of the other New World alphavirus VEEV (Figure 5H).

The arthritogenic alphaviruses ONNV, MAYV, and RRV showed a decreased viral replication in the HEK293T ΔSLC1A5 KO cell line (Figures 5I–K). For RRV also an effect was visible with the simultaneous deletion of CD147-SLC1A5 with both intermediate and low amounts of virus (Figure 5K). A trend of decreased viral replication of ONNV was observed in HEK293T ΔCD147 cells (Figure 5I). In the double KO cell line HEK293T ΔCD147-SLC1A5 no effect was visible for ONNV and MAYV (Figures 5I–J).

Altogether these data suggest a role for the CD147 protein complex in the replication process of the different lineages of CHIKV and related alphaviruses except for VEEV. However, the level of dependency on factors of the CD147 protein complex can differ between the different alphaviruses. For example, in the replication cycle of arthritogenic viruses ONNV, MAYV, and RRV mainly SLC1A5 seems to play a role. Also the effect in the double KO cell line HEK293T ΔCD147-SLC1A5 is not similar for all different alphaviruses.

### Protein Structure Alignment of CD147 and MXRA8

**FIGURE 6:**
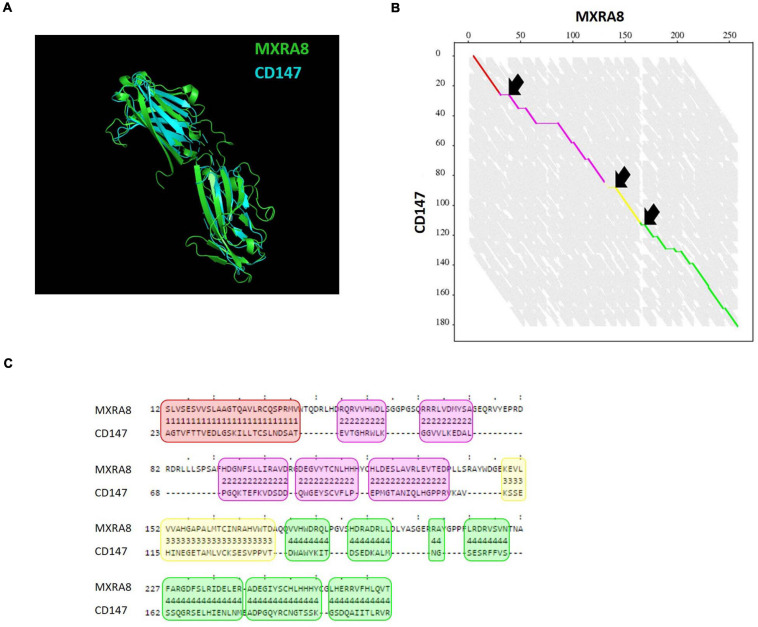
Protein structure comparison of CD147 and MXRA8. **(A)** Alignment of crystal structures of MXRA8 (PDB: 6JO7, green) and CD147 (PDB: 3B5H, blue) using the FATCAT algorithm (Ye and Godzik, 2004). **(B)** Alignment graph with four aligned fragment pairs (AFP) (red, purple, yellow, and green) and 3 twist (arrows). On the axes the length of the amino acid sequence of MXRA8 and CD147 is depicted. **(C)** Detailed alignment of the four AFPs shown in red, purple, yellow and green as in **(B)**. The amino acid sequence of MXRA8 and CD147 is shown.

MXRA8 was previously identified as a receptor for alphaviruses including CHIKV (Zhang et al., 2018, 2019; Basore et al., 2019; Song et al., 2019; Kim et al., 2020). Because both MXRA8 and CD147 isoform 2 carry two immunoglobulin-like domains and one transmembrane region, we wondered if any homology in amino acid sequence or protein structure could be found between these two proteins. Alignment of the amino acid sequences of MXRA8 and CD147 did not reveal any significant homology at that level. Subsequently, the FATCAT flexible algorithm was used for protein structure alignment (Ye and Godzik, 2004). FATCAT identifies multiple aligned fragment pairs (AFP) in the proteins to be compared, taking in account the flexibility of a protein structure (Figures 6B,C). AFPs are defined as a continuous fragment of the protein structure of protein A aligned to a continuous segment of the proteins structure of protein B. Interestingly, the superposition of MXRA8 and CD147 structures was found to be highly similar with a *p*-value of 8.28 × 10^–3^ (Figure 6A). Four AFPs were identified and three twists were introduced (Figures 6B,C). These data show that MXRA8 and CD147 have significantly similar structural topologies. However, we did not observe a preference of the CHIKV interacting residues present in MXRA8 to be located within the identified AFPs.

The Human Protein Atlas^2^ was used to compare the RNA expression profile of MXRA8 and CD147 both on a cell level as well as on tissue level. CD147 is highly expressed in diverse human cell and tissue types, including in relevant CHIKV target cells such as fibroblasts and endothelial cells. The expression of MXRA8 is largely restricted to fibroblasts. Overall the RNA expression of CD147 is much higher compared to MXRA8 expression, also in the CHIKV relevant target cells. Interestingly, MXRA8 is not expressed in HEK293T cells (used here; Supplementary Figure 7) or Hela cells both of which are highly susceptible to CHIKV infection (Zhang et al., 2018; Basore et al., 2019).

## Discussion

Over the last decade CHIKV rapidly spread to new areas around the world. The high attack rate combined with high morbidity caused by the chronic phase, make this an emerging infection with a significant economic impact, especially in low and middle income countries. The entry process is the initial rate-limiting step in the viral replication cycle. In order to enhance the understanding of the protein-protein interactions necessary in the CHIKV entry process, we conducted an affinity purification combined with mass spectrometry approach to identify unknown host factors that contribute to viral entry. This led to the identification of the CD147 protein complex as an entry dependency factor for CHIKV.

Our AP-MS approach for the identification of unknown entry factors was validated by the detection of previously identified binding factors for CHIKV like ATP1A1 and PHB in human cells (Wintachai et al., 2012; Ashbrook et al., 2016). As well as with the identification of the ATP5A1 subunit of ATP synthase, since ATP5B, another subunit of the ATP synthase complex, was previously identified as entry factor in mosquito cells (Fongsaran et al., 2014). We were however not able to identify the recently described alphavirus receptor MXRA8 as it is not expressed in HEK293T cells used in our study (Zhang et al., 2018). We proved that we were capable of producing functional envelope complexes with an internal Strep-tag capable of interacting with biological relevant host proteins. The envelope gene (E3-ST-E2-6k-E1) was carefully selected to not contain mutations that increase GAG dependency, which reduces the identification of proteins associated with GAG binding (Henrik Gad et al., 2012; Ashbrook et al., 2014; Silva et al., 2014; Weber et al., 2017).

In HEK293T ΔCD147-SLC1A5 cells not always an additional effect of the double KO on alphavirus replication was observed compared to the single KO cells. It is possible this is due to the low amount of residual CD147 expressed or the availability of alternative entry factors and/or pathways for a given alphavirus. Adjustments of the cells to the double KO could also have a different effect on the replication cycle of the various alphaviruses. Since the CD147 protein complex resides in the lipid rafts, KO of the protein complex might influence plasma membrane composition which could hamper efficient virus budding and thus lead to an accumulation of viral proteins in the cells that would be detected by immunofluorescence.

CD147, also called basigin or EMMPRIN (extracellular matrix metalloproteinase inducer), has two isoforms. Isoform 1 has three immunoglobulin-like domains and is only expressed in the retina. Isoform 2 has two immunoglobulin-like domains and is ubiquitously expressed. CD147 is highly glycosylated and is capable of recognizing other CD147 molecules in both *cis* and *trans* localization (Yu et al., 2008; Grass and Toole, 2016; Muramatsu, 2016). Its main function is inducing matrix metalloproteinases and it has pivotal roles in spermatogenesis, embryo implantation and neural network formation. CD147 is found to be overexpressed in many cancers (Grass and Toole, 2016). In addition, the protein associates with monocarboxylate transporters and is essential for their cell surface expression (Xu and Hemler, 2005; Muramatsu, 2016). It interacts with PfRh5 of *Plasmodium falciparum* and mediates erythrocyte invasion (Crosnier et al., 2011). After reintroduction of CD147 isoform 2 in the KO cell lines, mainly a low glycosylated form of CD147 was found to be expressed. Only a limited amount of high glycosylated CD147, similar to endogenous CD147 expressed in HEK293T cells, was produced. Since infectivity of CHIKV was increased upon reintroduction of CD147 in the CD147 KO cell lines, this suggests that either glycosylation of CD147 is not required for recognition by CHIKV or that only low levels of high glycosylated reintroduced CD147 are sufficient to restore susceptibility for CHIKV. SLC1A5 also known as ASCT2, is neutral amino acid transporter and often found to be overexpressed in cancer cells (Scalise et al., 2018). CD98 is a heavily glycosylated transmembrane protein which dimerizes with various amino acid transporters and regulates their membrane expression. Additionally CD98 interacts with the cytoplasmatic tails of integrin-β, amplifying integrin signaling (Cantor and Ginsberg, 2012).

In this study we only analyzed the effect of reintroducing CD147 isoform 2 on the replication cycle of CHIKV since this isoform is widely expressed. However an atypical and less frequent symptom associated with CHIKV is retinitis (Vairo et al., 2019). And since CD147 isoform 1 is solely expressed in the retina, it could be that also this isoform plays a role in alphavirus infection. The involvement of CD147 isoform 1, due to limited expression pattern, must be confined to the retina.

It has been shown that CD147 functions as a receptor for extracellular cyclophilins A and B (CypA and CypB) and mediates chemotactic activity of cyclophilins toward immune cells (Muramatsu, 2016; Kumar et al., 2019). The cyclophilin-CD147 interaction has a low-affinity and is also dependent on the presence of heparan sulfate proteoglycans (Yurchenko et al., 2010). Cyclophilins play a role in the entry of measles virus (Watanabe et al., 2010) and severe acute respiratory syndrome coronavirus (SARS) (Chen et al., 2005) since cyclophilins have been found to be incorporated in these virions. For example, the CypB-CD147 interaction is used as an entry pathway for measles virus in receptor negative cells (Watanabe et al., 2010). The binding of SARS is facilitated by the interaction between CypA and CD147 (Chen et al., 2005). However recently the spike protein of severe acute respiratory syndrome coronavirus 2 (SARS-CoV-2), very closely related with SARS and both using angiotensin-converting enzym 2 as receptor, was described to directly interact with CD147 (preprint; Wang et al., 2020). Contrary results were reported by *Shilts and Wright* who could not find a direct interaction between CD147 and SARS-CoV-2 (preprint;: Shilts and Wright, 2020). All together this suggests that both SARS and SARS-CoV-2 could use CD147 as entry receptor whether or not through cyclophilin binding. Although we identified CD147 after an affinity purification of the envelope proteins, a role of cyclophilins cannot be ruled out in alphavirus entry. However, so far no evidence exists for the presence of cyclophilins in alphavirus virions.

CD147 is expressed on the surface of erythrocytes proven by the fact that erythrocyte invasion by *Plasmodium falciparum* is mediated by CD147 (Crosnier et al., 2011). Except from our observation that depletion of CD147 negatively modulates the replication of multiple alphaviruses, CD147 mediated erythrocyte attachment could also possibly stabilize the viral particles in blood. Additionally alphavirus attachment to erythrocytes could also promote trans infections, previously described for human immunodeficiency virus 1 (Beck et al., 2009). A high viremia at symptom onset is observed with both alphaviruses as flaviviruses. And also the flavivirus West Nile virus is found to adhere to erythrocytes (Rios et al., 2007). Perhaps this high viremia is due to the capacity of these viruses to adhere to erythrocytes for stabilization.

Previously, MXRA8 was identified as an alphavirus receptor used by CHIKV, ONNV, MAYV, and RRV (Zhang et al., 2018). We found that when one or more factors of the CD147 protein complex were knocked out in HEK293T cells, viral infectivity was decreased for ONNV, MAYV, RRV, SINV, WEEV, and EEEV but not for VEEV. ONNV, RRV, MAYV, and SINV belong to the Old World alphaviruses and cause an arthritogenic disease similar to CHIKV whereas WEEV, EEEV, and VEEV are New World alphaviruses and cause an encephalitic disease. CD147 has been found to be upregulated in the synovial membrane, synoviocytes and macrophages present in the synovial fluid of rheumatoid arthritis patients (Konttinen et al., 2000; Tomita et al., 2002; Zhu et al., 2006). CHIKV like other arthritogenic alphaviruses, is in the chronic phase of the disease characterized by arthritis that can resemble in a high degree rheumatoid arthritis (Amaral et al., 2020). The different cell types shown to overexpress CD147 in rheumatoid arthritis patients are all susceptible to CHIKV (Sourisseau et al., 2007; Matusali et al., 2019). The usage of CD147 by the different arthritogenic alphaviruses could perhaps be a direct link to the observed arthritogenic symptoms.

The overall identity between the E proteins of the different alphaviruses probably does not readily explain the usage of the CD147 protein complex. The structural proteins of CHIKV have 85% identity with ONNV (Levinson et al., 1990; Khan et al., 2002), 60% with RRV (Khan et al., 2002; Fox et al., 2015), 44% with SINV, 48% with EEEV, 45% with VEEV, 43% with WEEV (Khan et al., 2002), and 56% with MAYV (only E2 protein) (Fox et al., 2015). The percentage identity with CHIKV of SINV, WEEV, EEEV, and VEEV is very similar but only SINV, WEEV, and EEEV seem to rely on the CD147 protein complex. Contrary to MXRA8, we observed a great effect of CD147 depletion on the replication of SINV and WEEV. CD147 also does not show any structural similarity with the previously identified SINV entry factor NRAMP2 (Rose et al., 2011). Overall we are not capable of determining the potential sensitivity to the CD147 protein complex of the different alphaviruses based on their primary amino acid sequence. No more than the interacting residues on the alphavirus envelope could explain differences in MXRA8 dependency between CHIKV strains or predict usage of MXRA8 by CHIKV, ONNV, RRV, and MAYV but not by SINV, VEEV, and WEEV (Zhang et al., 2018; Basore et al., 2019; Song et al., 2019). Determining and comparing the interaction residues and binding sites of the different alphaviruses with the CD147 protein complex could maybe help to resolve the distinctive factors that influence sensitivity to the CD147 protein complex.

Historically, WEEV is derived from a recombination event between a SINV-like virus and EEEV. The structural proteins of WEEV were obtained from the SINV-like virus and the non-structural proteins from EEEV (Forrester et al., 2012; Allison et al., 2015). We observed that following depletion of the CD147 protein complex, both replication of SINV and WEEV were severely hampered. WEEV and SINV have a very similar reaction to the depletion of the CD147 protein complex although being a New or Old world virus, respectively. Since WEEV has an envelope protein that is more similar to SINV, an arthritogenic alphavirus, than to the other encephalitic alphaviruses, this might be the reason that WEEV has a very similar dependency on the CD147 protein complex compared to SINV. Alphaviruses are divided in different antigenic complexes (Forrester et al., 2012; Powers, 2016). CHIKV, MAYV, RRV, ONNV are all part of the Semliki Forest virus complex while WEEV and SINV are part of the WEEV complex. VEEV and EEEV are classified into the VEEV complex and EEEV complex, respectively. Generally, we found dependency on members of the CD147 protein complex to be present in the Semliki Forest virus complex, the WEEV complex and the EEEV complex but not in the VEEV complex (Forrester et al., 2012). This suggests that the determining factors in alphaviruses for CD147 protein complex dependency were present in an ancestral alphavirus and was lost after the emergence of the VEEV complex. Dependency on the CD147 protein complex is also not unequivocally divided between New and Old world viruses or encephalitic and arthritogenic viruses.

An interesting observation is that CD147 protein and the recently identified CHIKV receptor MXRA8 (Zhang et al., 2018) have two extracellular immunoglobulin-like domains. *In silico* analysis found that both MXRA8 and CD147 isoform 2 have a similar tertiary protein structure while lacking significant amino acid sequence homology. This suggests that CHIKV can probably bind multiple host factors with large structural homology. Possibly the CHIKV envelope recognizes a specific structure rather than one specific protein for binding at the target cell membrane. The recognition of a common specific three-dimensional structure might allow CHIKV to quickly approach different receptors. The subsequent increased proximity between CHIKV and the receptors could accommodate the close interaction of the residues on the different receptors with several residues on E2. Since the different receptors do not share any amino acid homology, they could interact with the same or entirely different residues on E2 and/or E1. For the binding between CHIKV envelope proteins E1 and E2 and MXRA8 several interacting residues have been identified (Zhang et al., 2018; Basore et al., 2019; Song et al., 2019). The residues responsible for the interaction between the CD147 protein complex and the envelope proteins E1 and E2 are not yet identified. We generally observed a greater effect on alphavirus replication when several members of the CD147 protein complex were depleted. This could be due to an increased destabilization of the CD147 protein complex when multiple members are removed from the complex, or perhaps the recognition of the protein structure of CD147 helps the viral particle to more efficiently interact with an entry factor present in the CD147 protein complex. It is not excluded that CHIKV interacts with multiple members of the CD147 protein complex. Lastly, co-expression of different CHIKV receptors like CD147 and MXRA8 might lead to a synergistic effect and increased CHIKV susceptibility. *Zhang et al.* observed enhanced CHIKV infection when MXRA8 was overexpressed in HEK293T cells, which naturally lack MXRA8, suggesting the existence of synergistic roles for different CHIKV receptors (Zhang et al., 2018).

MXRA8 is not present on every cell type or cell line that can be readily infected by CHIKV (e.g., keratinocytes, HEK293T, HeLa) (Matusali et al., 2019) and is thus partly dispensable. The usage of multiple receptors with a similar structure could explain the broad tropism of CHIKV. Generally, CHIKV is capable of productively infecting fibroblasts, keratinocytes, melanocytes, epithelial and endothelial cells and to a lesser extent macrophages (Sourisseau et al., 2007; Matusali et al., 2019). We also could not completely block CHIKV infection in stable CD147 KO cells, although replication was suppressed to a similar degree as when a key component of CME was removed. Together these findings suggest that the entry of CHIKV is a multi-route process, where the virus can employ several receptors as well as different endocytosis pathways (e.g., CME and macropinocytosis; Bernard et al., 2010; Lee et al., 2019).

In conclusion, CD147 is a highly glycosylated protein and broadly expressed on human cell types making it is an interesting target for binding by pathogens. We present strong evidence for the involvement of the CD147 membrane protein complex in the entry process of CHIKV and related alphaviruses. The strong structural similarity of CD147 and MXRA8, suggest that alphavirus entry is a promiscuous process driven by recognition of structural motifs rather than binding to a single receptor molecule.

## Data Availability Statement

The original contributions presented in the study are included in the article/Supplementary Material, further inquiries can be directed to the corresponding author/s.

## Author Contributions

LD, KB, and KA: designed the experiment, data analysis, and wrote manuscript. LD, SC, KV, and KB: performed experiments (except mass spectrometry). SD, MD, and DD: mass spectrometry analysis. KB, KA, and XV: supervision. All authors contributed to the article and approved the submitted version.

## Conflict of Interest

The authors declare that the research was conducted in the absence of any commercial or financial relationships that could be construed as a potential conflict of interest.

## Footnotes

1 https://chopchop.cbu.uib.no/

2 http://www.proteinatlas.org
