# Supplementary material for: The CD147 Protein Complex Is Involved in Entry of Chikungunya Virus and Related Alphaviruses in Human Cells
Source: Front Microbiol. 2021 Feb 25;12:615165. doi: 10.3389/fmicb.2021.615165 (PMC7946996; doi:10.3389/fmicb.2021.615165)
Supplement: Supplementary file 1 [file Data_Sheet_1.docx]

Supplementary Material

# Supplementary Figures and Tables

## Supplementary Figures

**Sup Fig 1 Linearity of reporter virus luciferase signal.**. Dilution series of the virus stock of the reporter CHIKV virus carrying a nanoluciferase gene in nsp3. The values at the lowest dilution point (1x10^-1^) were limited by the detection limit of TriStar LB 941 Multimode Microplate Reader (Berthold Technologies).


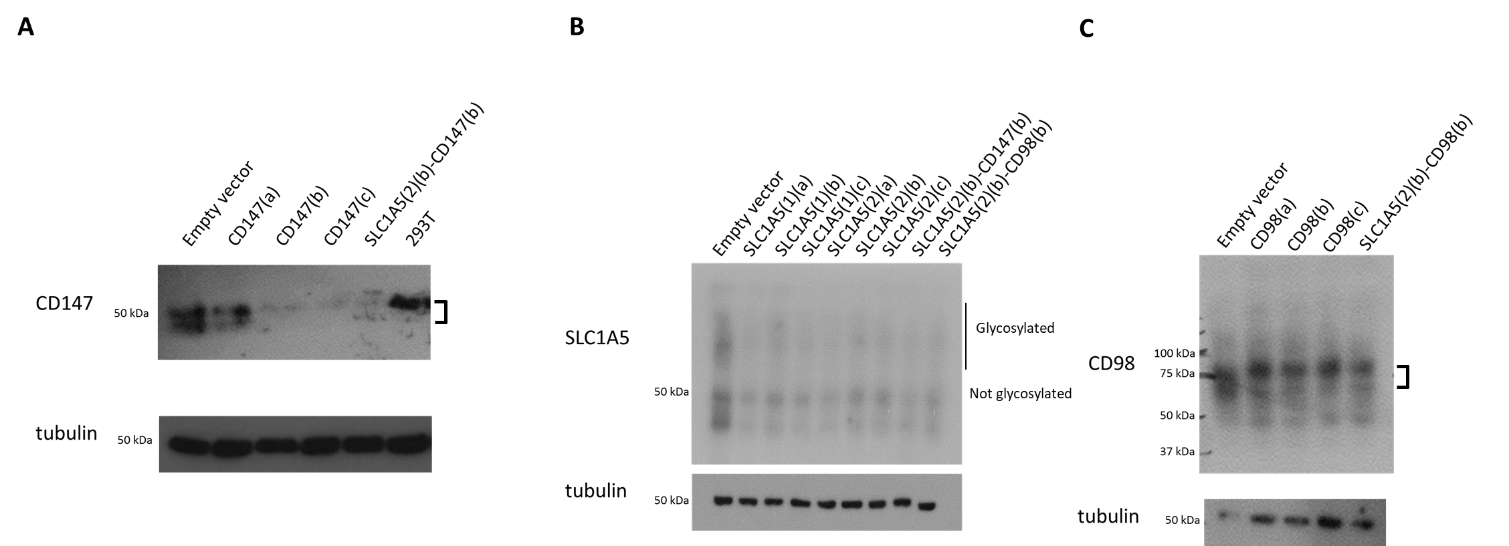


**Sup Fig 2 Knockout efficiency after transient transfection.** Lysate of HEK293T cells transfected with different gRNA’s and selected with puromycin. Lysate of empty vector cells was used as positive control. Western blot of CD147, SLC1A5 and CD98.


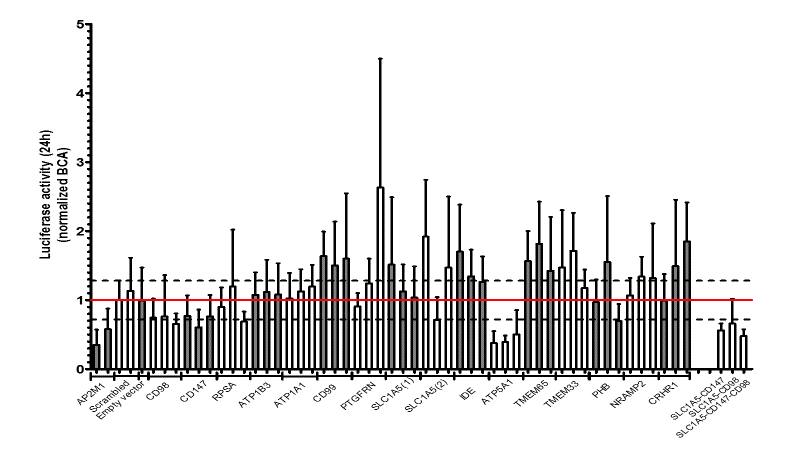


**Sup Fig 3 CRISPR/Cas9 knockout screen of identified human protein interactors.** HEK293T cells were transiently transfected with three different gRNA’s per gene whereafter transfected cells were selected with puromycin. As a positive control AP2M1 and as a negative control Scrambled and Empty vector, Cas9 vector not containing gRNA, were included. Data was normalized for scrambled. Dashed lines depict SD for scrambled. Infection with reporter virus containing nanoluciferase for 24h (entry dependent). Mean ± SD is depicted (n = at least 6)


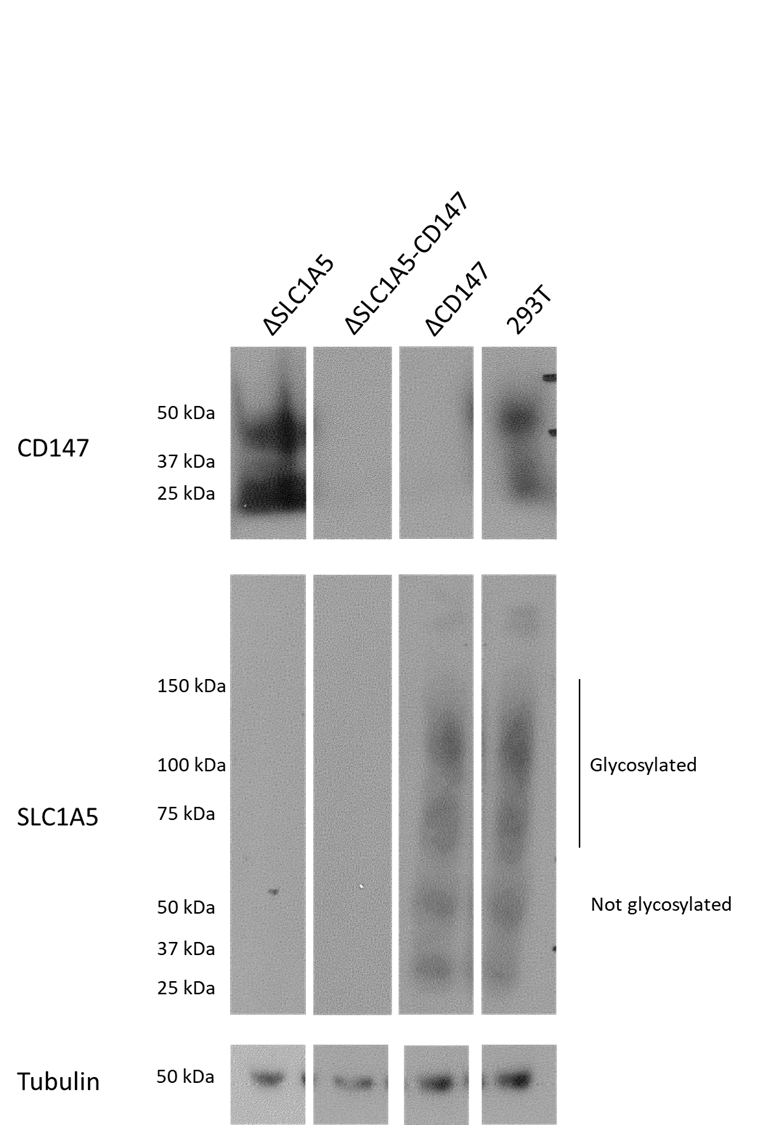


**Sup Fig 4 Knockout cell lines.** Western blot for total cellular levels of CD147, SLC1A5 and tubulin. Lysate of HEK293T cells was used as positive control.

**Sup Fig 5 CD147 membrane expression on knockout cell lines**. Membrane expression of CD147 on the stable KO cell lines analyzed using flow cytometry. Percentage CD147-positive cells is depicted. Mean ± SD is shown (n = 3; 1 independent experiment with 3 technical replicates)


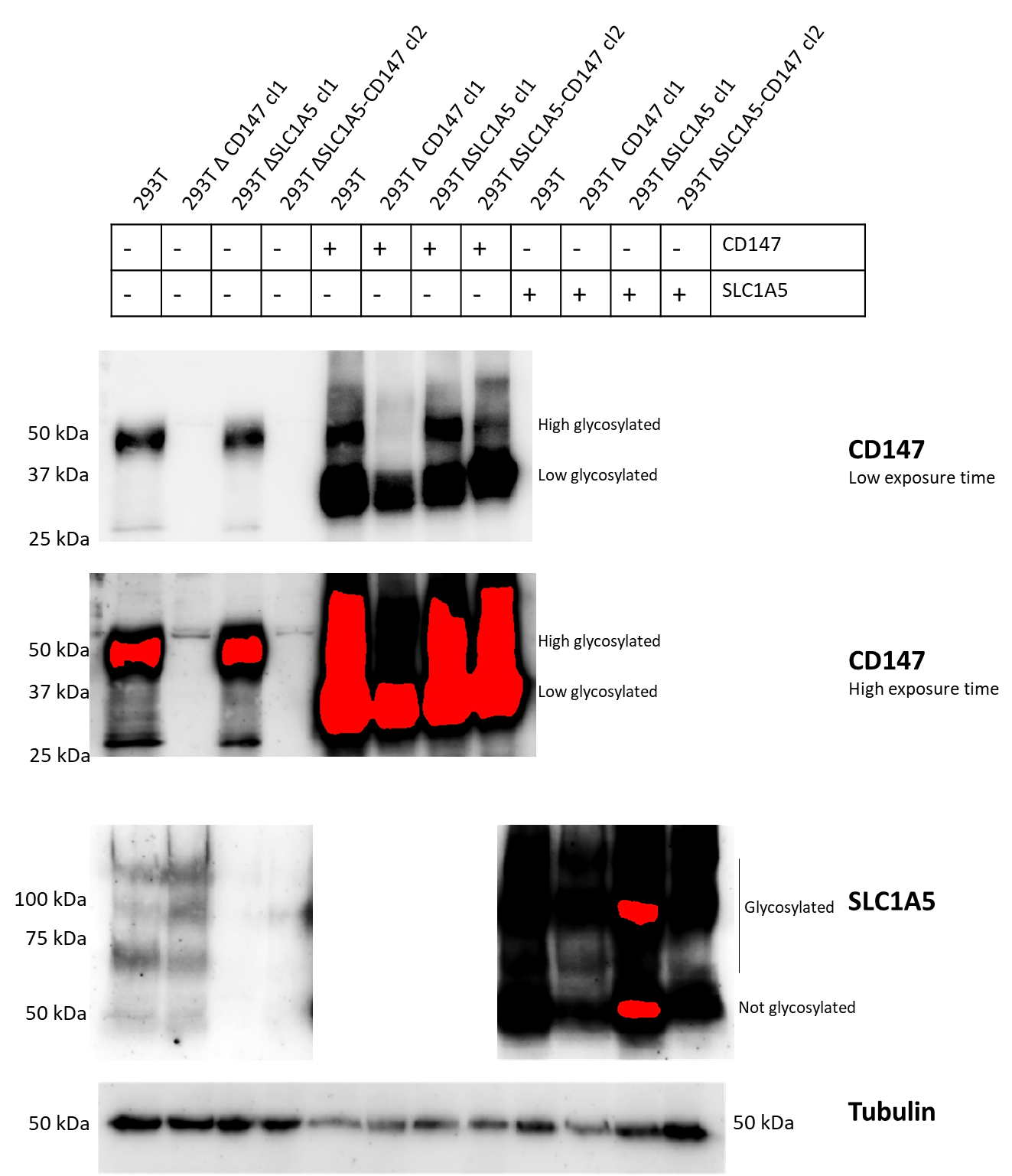


**Sup Fig 6 CD147 and SLC1A5 protein expression before and after reintroduction.** Cell lysate of HEK293T cells and HEK293T KO cell lines for CD147, SLC1A5 and SLC1A5-CD147 was used. Cells were harvested and lysed 48h after transfection with overexpression constructs for CD147 and SLC1A5. High and low exposure blots for CD147 are depicted. Western blot of CD147, SLC1A5 and tubulin is shown.


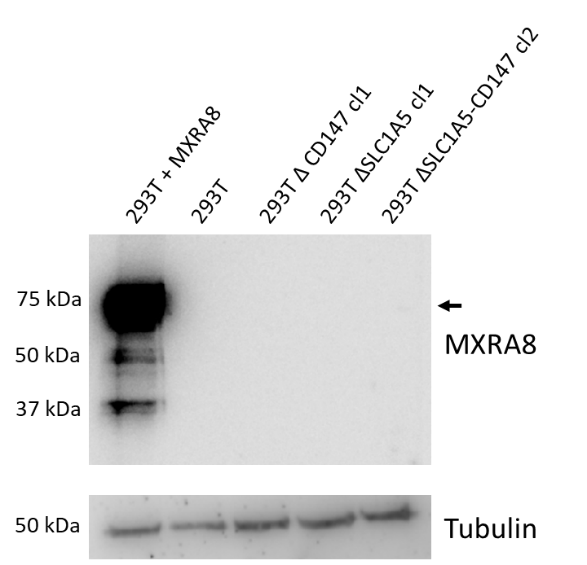


**Sup Fig 7 MXRA8 expression in HEK293T cells.** Western blot for cellular levels of MXRA8 and tubulin. Cell lysate of HEK293T cells and HEK293T KO cell lines for CD147, SLC1A5 and SLC1A5-CD147 was used. As a positive control MXRA8 fused with DsRed was transiently expressed in HEK293T cells.
